# Comparative analysis of spatial-temporal patterns of human metapneumovirus and respiratory syncytial virus in Africa using genetic data, 2011–2014

**DOI:** 10.1186/s12985-021-01570-8

**Published:** 2021-05-29

**Authors:** John W. Oketch, Everlyn Kamau, James R. Otieno, Anthony Mwema, Clement Lewa, Everlyne Isoe, D. James Nokes, Charles N. Agoti

**Affiliations:** 1 Kenya Medical Research Institute (KEMRI) -Wellcome Trust Research Programme, Kilifi, Kenya; 2 School of Pure and Applied Sciences, Pwani University, Kilifi, Kenya; 3 School of Life Sciences, and Zeeman Institute for Systems Biology and Infectious Disease Epidemiology Research (SBIDER), University of Warwick, Coventry, UK

**Keywords:** Human metapneumovirus, Respiratory syncytial virus, Phylogeographic analysis, Spatial-temporal

## Abstract

**Background:**

Human metapneumovirus (HMPV) and respiratory syncytial virus (RSV) are leading causes of viral severe acute respiratory illnesses in childhood. Both the two viruses belong to the *Pneumoviridae* family and show overlapping clinical, epidemiological and transmission features. However, it is unknown whether these two viruses have similar geographic spread patterns which may inform designing and evaluating their epidemic control measures.

**Methods:**

We conducted comparative phylogenetic and phylogeographic analyses to explore the spatial-temporal patterns of HMPV and RSV across Africa using 232 HMPV and 842 RSV attachment (G) glycoprotein gene sequences obtained from 5 countries (The Gambia, Zambia, Mali, South Africa, and Kenya) between August 2011 and January 2014.

**Results:**

Phylogeographic analyses found frequently similar patterns of spread of RSV and HMPV. Viral sequences commonly clustered by region, i.e., West Africa (Mali, Gambia), East Africa (Kenya) and Southern Africa (Zambia, South Africa), and similar genotype dominance patterns were observed between neighbouring countries. Both HMPV and RSV country epidemics were characterized by co-circulation of multiple genotypes. Sequences from different African sub-regions (East, West and Southern Africa) fell into separate clusters interspersed with sequences from other countries globally.

**Conclusion:**

The spatial clustering patterns of viral sequences and genotype dominance patterns observed in our analysis suggests strong regional links and predominant local transmission. The geographical clustering further suggests independent introduction of HMPV and RSV variants in Africa from the global pool, and local regional diversification.

**Supplementary Information:**

The online version contains supplementary material available at 10.1186/s12985-021-01570-8.

## Introduction

Human metapneumovirus (HMPV) and respiratory syncytial virus (RSV) are leading viral respiratory pathogens that cause seasonal epidemics of acute respiratory tract illness and are responsible for a significant fraction of childhood pneumonia [1]. A multi-country study named PERCH (Pneumonia Etiology Research for Child Health), undertaken between 2011 and 2014 in Africa and Asia reported RSV as the leading cause of pneumonia in children aged under five years accounting for at least 31% of the aetiological distribution [2]. In the same study, HMPV accounted for 5% of the aetiological distribution. The current study presents a molecular-epidemiological analysis of samples collected by the PERCH study from the five African counties, i.e., Kenya, South Africa, Zambia, Mali and The Gambia.

RSV and HMPV infections present with overlapping clinical and epidemiological profiles [3, 4]. Following infection with either virus, the clinical presentation can range from asymptomatic infection to mild upper respiratory tract illness to severe lower respiratory tract disease. Further, these clinical features are also observed with several other respiratory viruses e.g. influenza and human coronaviruses [3, 5]. Both HMPV and RSV infect persons across all ages but severe disease is majorly limited to infants and young children, the vulnerable adult populations (the elderly, immunocompromised and persons with cardiopulmonary co-morbidities) [4, 6, 7]. Re-infection with these viruses occurs throughout life probably due to incomplete immunity that wanes over time combined with ongoing antigenic variation in key viral immune epitopes which may support antibody escape [8, 9]. RSV and HMPV seasonal patterns are observed to frequently overlap. In temperate climatic regions they tend to peak in cold seasons while in the tropics the association of peak transmission months and weather patterns has been inconsistent [10]. Transmission of these two viruses is primarily via direct inhalation of infected droplets or indirect via fomites (contaminated objects or surfaces) contacts [11–13].

The two viruses belong to *Pneumoviridae* family and share several genomic features [14]. HMPV genome is about 13 kb encoding eight genes (3′N–P–M–F–M2–SH–G–L5′) while RSV genome is about 15 kb encoding ten genes (3′NS1-NS2-N-P-M-SH-G-F-M2-L5) thus HMPV has a different gene order and lacks non-structural proteins NS1 and NS2 [14]. For both RSV and HMPV, the attachment glycoprotein (G) gene is the most genetically variable region across their entire genomes and is commonly used to discriminate genetic variants [15, 16]. HMPV is classified into two groups, A and B, based on antigenic and genetic differences mainly in the fusion (F) and attachment (G) glycoprotein genes [16]. Based on the genetic differences, the two groups are further classified into four subgroups, A1, A2 (group A) and B1 and B2 (group B) [16]. Subgroup A2 is the most genetically diverse and is further divided into sub-lineages A2a and A2b [17, 18]. Additionally, there are two distinct clades within A2b, A2b1 and A2b2 [18].Similarly, RSV is classified into two groups (A and B) that are both antigenically and genetically distinct [19]. The two groups are further divided into multiple genotypes based on nucleotide differences within the RSV G gene [20]. Clinically, there is no difference in disease severity between the subgroups for both HMPV and RSV [5, 21]. Epidemiological studies have shown that multiple HMPV and RSV subtype/genotypes can co-circulate during epidemics both locally and globally, implying fast and widespread dispersal HMPV and RSV variants once they arise [22, 23]. The dominant subgroup/genotype can also vary based on year and location [15, 23].

It is unclear whether HMPV and RSV share geographic spread patterns. Although this can be investigated using sequence and spatial-temporal data, such data is scarce and there is asynchronous sampling in time and space, especially in Africa [24]. As a result, the origins and interconnectedness of RSV and HMPV epidemics across many global locations including Africa is not well understood. Integrating pathogen sequence data with other data e.g. spatial-temporal data allows reconstruction of transmission histories necessary for tracing of epidemiological linkages especially when there is limited case surveillance and tracing [25, 26]. Both HMPV and RSV are undergoing continuous genetic sequence evolution leading to occasional emergence of novel genotypes [27–29] thus understanding their geographic spread could help inform interventions in future epidemics. Here, we report comparative phylogenetic analysis of HMPV and RSV sequence data collected between 2011–2014 across five African countries (Kenya, Mali, Gambia, South Africa and Zambia) located in different sub-regions (East, West and South). Our study provides an initial view of RSV and HMPV phylogeography across Africa detailing their overall spatial-temporal transmission patterns within the continent in relation to the rest of the world.

## Materials and methods

### Study samples

The study analyzed nasopharyngeal (NP) flocked swab or a combination of nasopharyngeal swab and oropharyngeal (OP) swabs positive for HMPV and RSV. The samples were identified during the PERCH study [2, 30, 31] conducted between August 2011 and January 2014 from 5 African countries (The Gambia, Zambia, Mali, South Africa and Kenya), Table 1 and Additional file 1. A single hospital site, backed by well-defined catchment areas of known population size, was selected in each country [30]. Site characteristics for each country are reported in [30, 31]. Cases (hospital admissions) and controls (persons attending outpatient facilities for mild illness or vaccination) were selected within the defined catchment areas. Cases included children aged between 28 days and 59 months with severe or very severe pneumonia [30, 31]. Controls were randomly enrolled regardless of the respiratory symptoms and matched to cases by location and age group (1 to < 6 months, 6 to < 12 months, 12 to < 24 months, and 24–59 months of age) [2, 31]. Written informed consent was obtained from the parent or a guardian of the enrolled children.

**Table 1:** Virus positive by site and number sequenced

Site	Enrollment date	No. of samples	Cases		Controls		Total sequenced
			No. of cases	No. sequenced	No. of controls	No. sequenced	
*(A) HMPV*							
Gambia	November 2011–October 2013	46	37	32	9	9	41
Kenya	August 2011–November 2013	62	50	50	13	8	58
Mali	January 2012–January 2014	46	39	34	7	6	40
South Africa	August 2011–August 2013	77	55	44	22	14	58
Zambia	October 2011–October 2013	47	39	30	8	5	35
Totals		278	200	190	59	42	232

Site	Enrollment date	No. of samples	Cases		Controls		Total sequenced
			No. of cases	No. sequenced	No. of controls	No. sequenced	
*(B) RSV*							
Gambia	November 2011–October 2013	117	113	97	4	2	99
Kenya	August 2011–November 2013	263	251	251	12	12	263
Mali	January 2012–January 2014	182	154	138	28	20	158
South Africa	August 2011–August 2013	260	232	208	28	22	230
Zambia	October 2011–October 2013	112	94	82	18	10	92
Totals		934	844	776	90	66	842

Total number of HMPV and RSV positive samples collected between August 2011 and January 2014 from 5 African countries. Panel A: Total number of HMPV sequences stratified by cases and controls, and total sequenced. Panel B: Total number of RSV sequences stratified by cases and controls, and total sequenced

HMPV, human metapneumovirus; RSV, respiratory syncytial virus

The present study was approved by the Scientific and Ethical Review Unit that sits at KEMRI in Nairobi (SERU# 3443) and the PERCH Committee (http://www.jhsph.edu/ivac/resources/perch-background-and-methods/).

### Laboratory methods

Viral RNA was extracted using QIAamp Viral RNA Minikit (Qiagen, Germany) following the manufacturer’s instructions. Reverse transcription and PCR amplification of the HMPV and RSV G genes followed protocols that have been reported elsewhere [23, 32]. Briefly, HMPV PCR primers amplified full G gene, approximately 700 bp in a one-step reverse transcription (RT) PCR using Qiagen kit. HMPV subgroup specific primers were used (Additional file 2) and have been reported in [23]. Thermocycling conditions were set at: 50 °C for 30 min, 95 °C for 15 min, 38cycles of 94 °C for 1 min, 53 °C for 1 min, 72 °C for 1 min, and a final extension of 10 min at 72 °C [23]. For RSV, a two-step PCR protocol was employed. The first-round amplification was performed using Qiagen one-step RT-PCR kit, and the second-round nested PCR performed using Qiagen TaqMan PCR kit mastermix. Thermocycling conditions and primers used have been reported elsewhere [32] RSV cross-group primers were used in both first and second-round PCR steps, and subgroup specific primers included in sequencing (Additional file 2). Amplified fragments were sequenced in both forward and reverse strands using the BigDye Terminator v1.3 chemistry on ABI 3130xl. The sequenced contigs were assembled using Sequencher v5.4.6 (Gene Codes Corporation). For Kenyan samples, the sequences have been previously reported in different studies under the accession numbers listed in Additional File 3. For The Gambia, Mali, South Africa and Zambia the sequences are reported in this study under the accession numbers shown in Additional file 3 for each country. The GenBank accession numbers of the contemporaneous sequences analysed in this study are also listed in Additional file 3.

### Sequence analysis

Sequences were aligned using MAFFT v7.407 [33] and manually curated in AliView v1.26 [34]. Pairwise genetic distances were calculated in MEGA v7. 0.2. software [35] under the Maximum Composite Likelihood method to assess the genetic diversity between sequences within the groups.

### Phylogenetic and phylogeographic analysis

The best fitting nucleotide substitution and site heterogeneity models were determined using ModelFinder [36] in IQ-TREE v1.6.11 [37]. Phylogenetic trees were constructed using the Maximum Likelihood (ML) approach in IQ-TREE v1.6.11. Branch support was evaluated by bootstrapping. HMPV and RSV subgroups/genotypes were confirmed if sequences clustered with known subgroups or prototype sequences of HMPV and RSV retrieved from GenBank (Additional file 3).

Phylogeographic analyses were done in BEAST v1.10.4 [38]. First, preliminary analysis was done to test for temporal signal and to identify the best coalescent demographic model. The temporal signal in the sequence data i.e. a root-to-tip divergence of genetic distance against the year of sampling was assessed using TempEst software v1.5.3 [39]. Four coalescent tree priors i.e. constant population size, exponential growth, Bayesian skyline plot and Bayesian Gaussian Markov Random Field (GMRF) skyride plot were tested under an uncorrelated lognormal relaxed molecular clock, and path sampling and stepping-stone analyses carried out to estimate marginal likelihoods [40]. The marginal likelihood measures the average fit of a model to the data [38]. Lower marginal likelihoods indicated weak evidence against the competing model. The Markov Chain Monte Carlo (MCMC) chains convergence [effective sample size (ESS) > 200] were evaluated in TRACER v1.7.1 [41]. The best combination of uncorrelated lognormal relaxed molecular and demographic models was selected for subsequent analysis.

A discrete trait representing geographical location was assigned to each sequence: Western Africa (Mali, Gambia), Eastern Africa (Kenya) and Southern Africa (South Africa and Zambia). To attain high spatial resolution, the country of sampling was also assigned to sequences. Viral dispersal patterns between locations were inferred using the Bayesian symmetric discrete trait evolution model with Bayesian stochastic search variable selection (BSSVS) procedure, implemented in BEAST v1.10.4. software. The symmetric diffusion model infers ancestral reconstruction using the standard continuous-time Markov chain (CTMC), in which the transition rates between locations are reversible [25]. MCMC chains were run for at least 200 million generations sampling every 20,000 steps.

Contemporaneous sequences with known collection date were retrieved from GenBank (Additional file 3) for global phylogeographic analysis. Due to the scarcity of HMPV G gene sequences, 714 sequences collected from 20 countries between 2000 and 2018 were included. For RSV, sequences collected a year before (2010) and after (2015) our study were analysed to place our data into immediate context. A total of 1810 sequences from 28 different countries were retrieved (Additional file 3). Both the continent and the country of sampling were assigned sequences as discrete traits. The global phylogeographic analysis was carried out under the symmetric diffusion model. The BEAST trees were summarised using Tree annotator v2.6.0 [42] after the removal of 10% burn-in. Maximum clade credibility trees (MCC) were visualized in FigTree v1.4.4. (http://tree.bio.ed.ac.uk/software/figtree/). Significant migration events between discrete locations were determined using the Bayes factor (BF) [40] and summarized using SpreaD3 v0.9.7.1 software [43] after discarding 10% burn-in. BF ≥ 1000 indicated very strong support, 10 ≤ BF ≤ 1000 strong support, and 3 ≤ BF ≤ 10 supported viral migration pathways.

## Results

### HMPV and RSV subgroup detection and temporal patterns

**Fig. 1:**
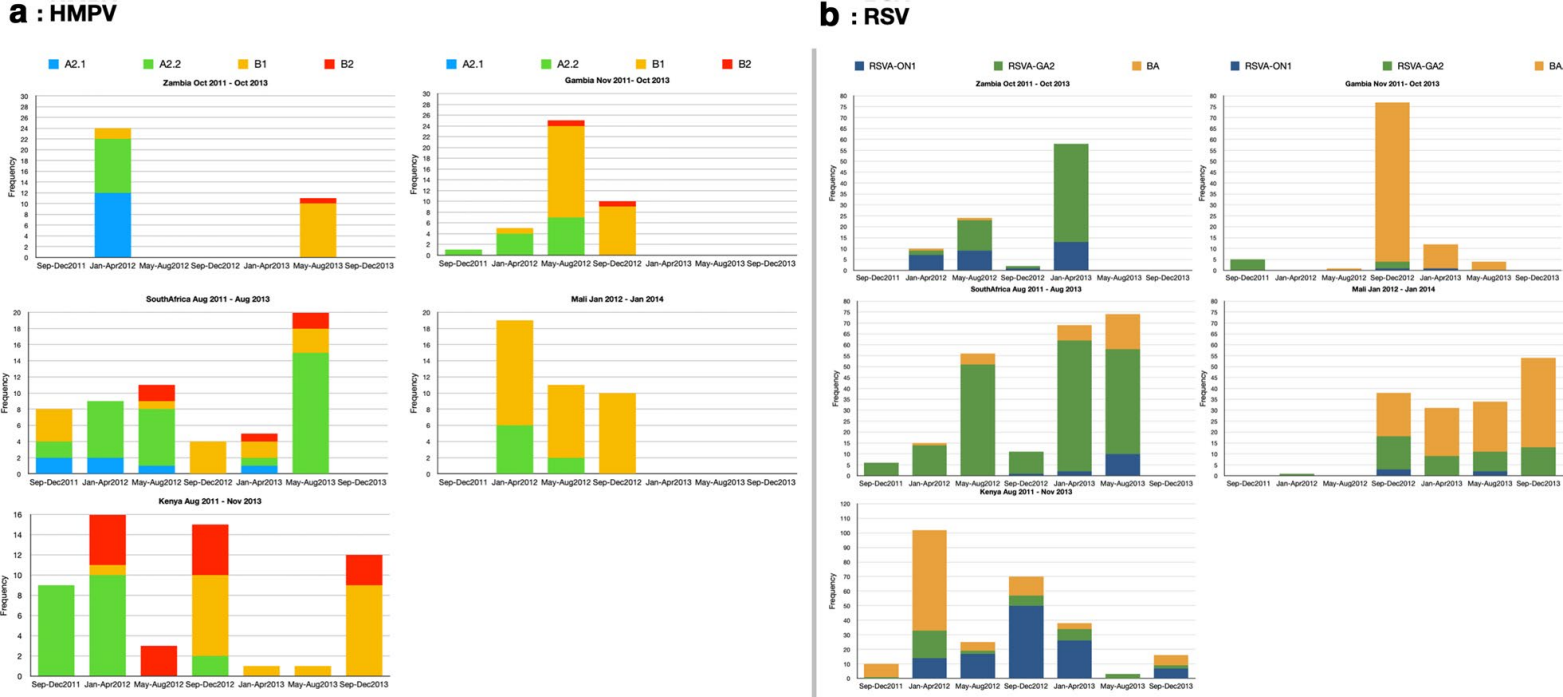
HMPV and RSV subgroup prevalence and temporal patterns derived from G gene sequence data collected from Kenya, Mali, Gambia, South Africa and Zambia. **a** HMPV temporal patterns. **b** RSV temporal patterns

In total, 232 HMPV G gene sequences were obtained of which 44% (102/232) belonged to subgroup A2 and further clustered into sub-lineages A2.1 (18%, 18/102) and A2.2 (82%, 84/102) (Additional file 4). Sub-lineage A2.2 further clustered into two distinct clades, A2.2.1 (35/84) and A2.2.2 (49/84) (Additional file 4). There were no subgroup A1 viruses. Among the sequenced HPMV strains, 56% (130/232) belonged to HMPV group B, of which 82% (107/130) and 18% (23/130) were subgroup B1 and B2, respectively. Multiple subgroups co-circulated in each country (Fig. 1a). Notably, A2.1 viruses were only identified in South Africa and Zambia. HMPV subgroup temporal patterns in Mali mirrored those in The Gambia (Fig. 1a). For RSV, a total of 842 sequences were analysed. Based on the RSV G gene phylogeny (Additional file 4), there were 509/842 (60%) RSVA and 333/842 (40%) RSVB sequences. All RSV B sequences belonged to the genotype BA. Among RSV A, 32% (163/509) were genotype ON1, and 68% (346/509) were genotype GA2. Similar to HMPV, multiple RSV genetic groups co-circulated within epidemics (Fig. 1b). Similar genotype dominance patterns were observed between Mali and Gambia, South Africa and Zambia, and were all different from Kenya (Fig. 1b).

### HMPV Intra-country genetic diversity

Only subgroup B1 viruses were detected in high frequencies in all the five countries and were analysed for intra-country diversity (Table 2). ML trees were reconstructed independently for each country. At least two well supported (bootstrap value > 95%) phylogenetic clades were observed in each country (Additional file 5). Sequences from different within-country sampling locations were mixed within the phylogenetic clusters suggesting rapid spread of HMPV variants within each country. Sequences from cases and controls were mixed within the clades (Additional file 5).

**Table 2:** HMPV and RSV subgroup detection patterns

Country	A2.1	A2.2	B1	B2	Total
*(A) HMPV subgroup detection*					
Kenya	0	21	21	16	58
Gambia	0	12	27	2	41
Mali	0	8	32	0	40
South Africa	6	33	15	4	58
Zambia	12	10	12	1	35
Total	18	84	107	23	232

Country	RSVA_ON1	RSVA_GA2	RSVB_BA	Total
*(B) RSV subgroup detection*				
Kenya	114	42	107	263
Gambia	2	8	89	99
Mali	5	47	106	158
South Africa	13	188	29	230
Zambia	29	61	2	92
Total	163	346	333	842

Total number of HMPV and RSV sequences obtained from samples collected between August 2011 and January 2014 from the 5 African countries. Panel A: Total number of HMPV sequences obtained by HMPV subgroup for each study site. Panel B: Total number of RSV sequences obtained by RSV subgroup for each study site

HMPV, human metapneumovirus; RSV, respiratory syncytial virus

### HMPV spatial origins and dispersal patterns in Africa

**Fig. 2:**
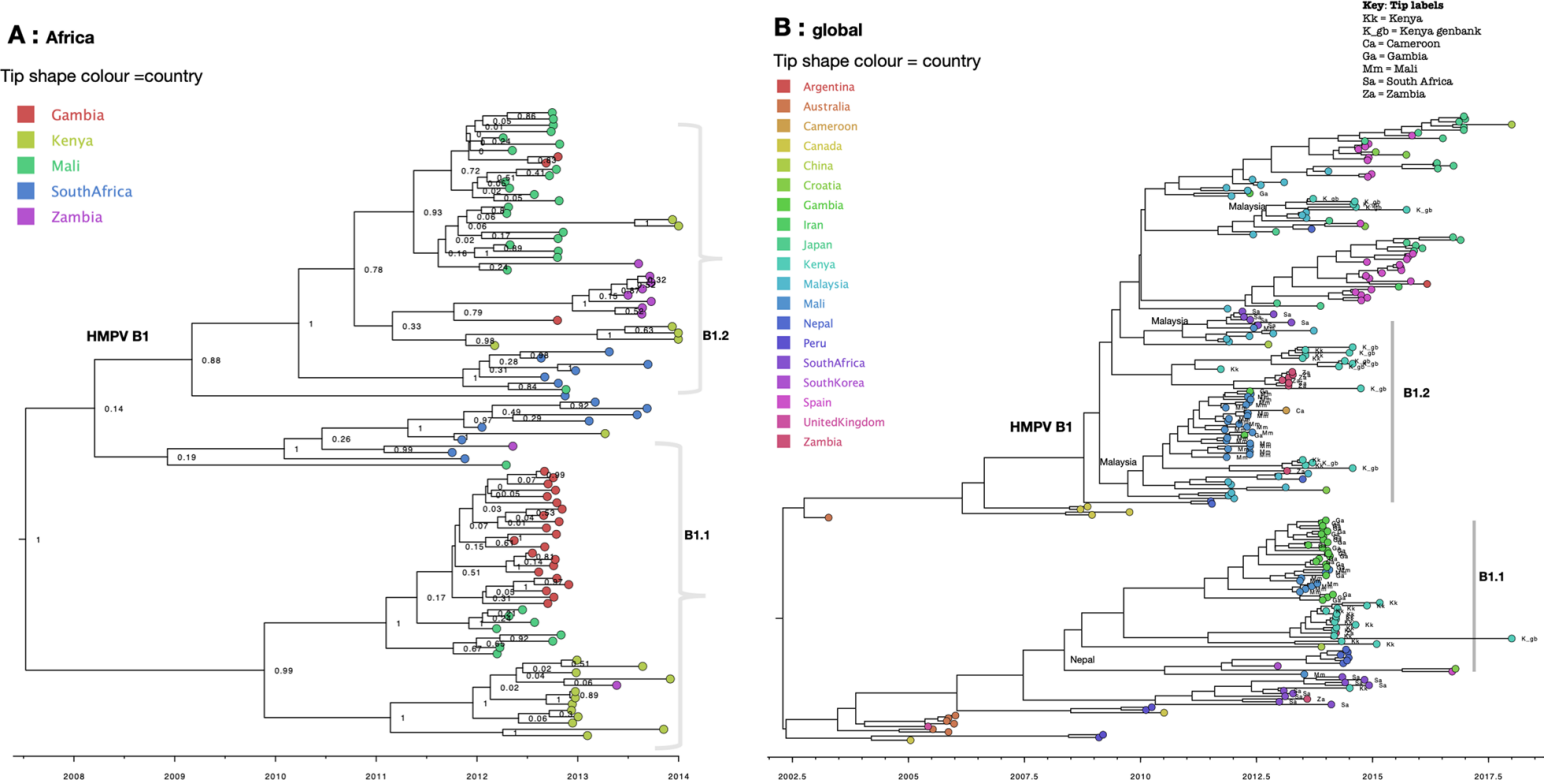
Time-scaled maximum clade credibility (MCC) trees of HMPV B1 G gene sequences. **a** Phylogenetic tree of B1 sequences obtained from Africa collected between August 2011 to January 2014. Tip shapes are coloured by country of sampling. **b** Phylogeny of HMPV B1 G gene sequences obtained from Africa and GenBank collected between 2000 to 2018. Tree tips are coloured by country of sampling. Geographic locations considered are shown in the figure key. Any sequences from Kenya, Mali, Gambia, South Africa and Zambia obtained from GenBank and collected beyond the study period are indicated with a suffix gb. Clades containing African sequences were labelled to reflect genetic clusters observed on the continental (Africa) B1 phylogeny—highlighted by grey vertical bars. African sequences are also indicated with tip labels. The most probable location of ancestral sequence at the branches leading to each African monophyletic clade is shown next to the nodes. Only ancestral locations with posterior probability support of > 70% were indicated

B1 sequences clustered into two major phylogenetic clades, numbered B1.1 and B1.2 (Fig. 2a). Sequences from the same geographical region, i.e. West Africa (Mali and Gambia), East Africa (Kenya) and Southern Africa (South Africa and Zambia) closely clustered together (Fig. 2a). On the global MCC tree the two clades (B2.1 and B2.2) were placed into two major clades alongside global sequences, suggesting that at least two distinct B1 variants were in circulation (Fig. 2b). The two variants reflect the genetic clusters that were observed on country-specific ML phylogenies above (Additional file 5). Clade B1.1 clustered closely with sequences from Nepal, and a few from Croatia and Spain. Clade B2.2 clustered closely with sequences from Malaysia. Although B1 sequences from Africa were interspersed with global sequences, they mostly clustered together. Of note, 81% (178/228) of B1 sequences were from Africa and Asia, making it difficult to assess viral introductions from unsampled locations.

**Fig. 3:**
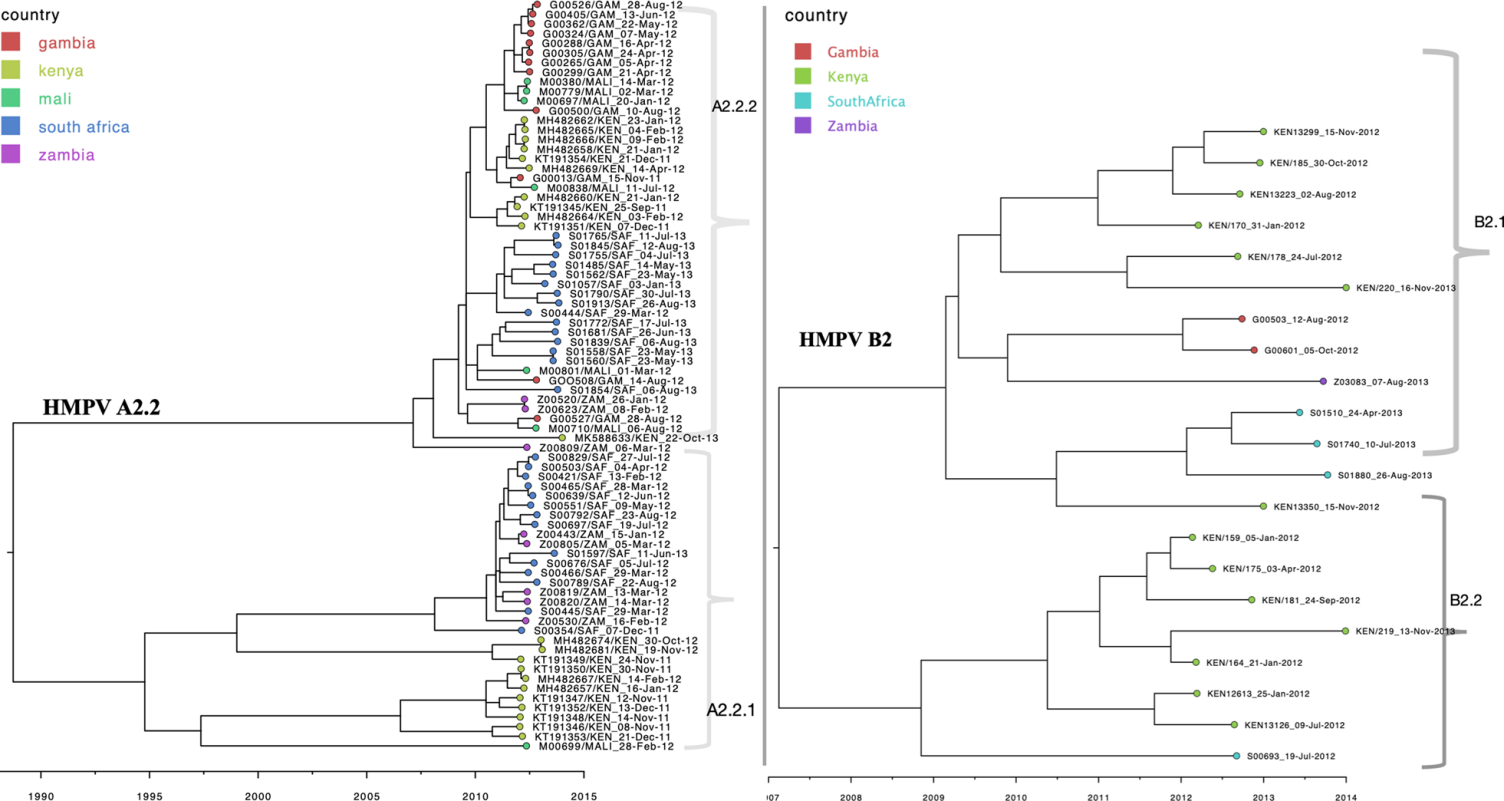
Time-scaled maximum clade credibility (MCC) trees constructed using HMPV A2.2 (**a**) and HMPV B2 (**b**) G gene sequences obtained from Africa, collected between August 2011 and January 2014. Tip shapes are coloured by country of sampling

**Fig. 4:**
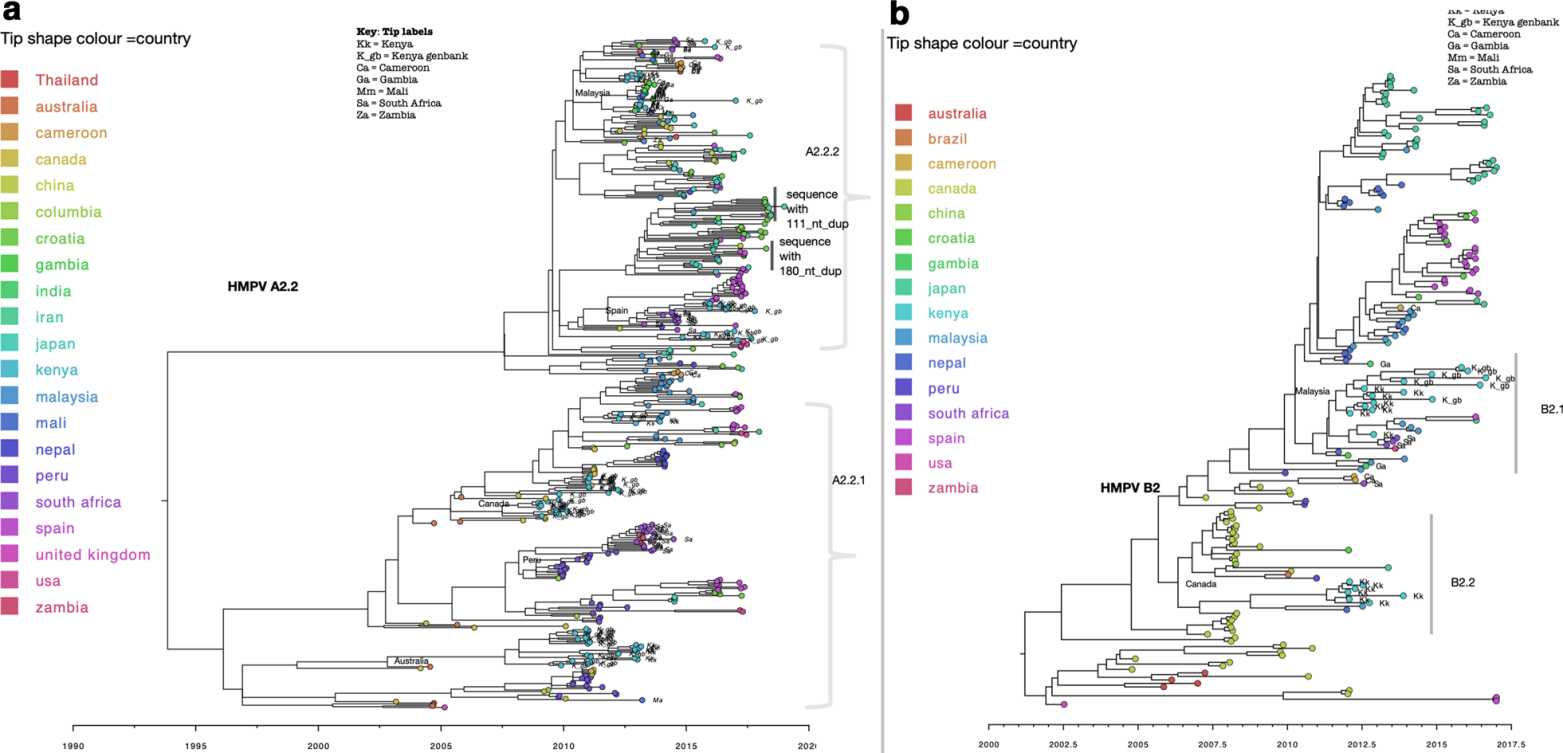
Time-scaled maximum clade credibility (MCC) trees constructed using HMPV A2.2 (**a**) and HMPV B2 (**b**) G gene sequences obtained from Africa and GenBank, collected between 2000 to 2018. Tree tips are coloured by country of sampling. Geographic locations considered are shown in the figure key. Any sequences from Kenya, Mali, Gambia, South Africa and Zambia obtained from GenBank and collected beyond the study period are indicated with a suffix _gb. African sequences are indicated with tip labels. The most probable location of ancestral sequence at the branches leading to each African monophyletic clade is shown next to the nodes. Only ancestral locations with posterior probability support of > 70% were indicated

Consistent with B1 MCC phylogenies of A2.2 and B2 African sequences showed at least two circulating variants for each subgroup (Fig. 3). Sequences from South Africa and Zambia clustered together. Similarly, sequences from Gambia and Mali clustered more closely among themselves, indicating an epidemiological linkage between neighbouring countries and separate introductions of HMPV variants in Africa. For A2.2, sequences clustered into two major clades numbered A2.2.1 and A2.2.2 (Fig. 3a). Similarly, B2 viruses clustered at least into two major clades, B2.1 and B2.2 (Fig. 3b). Clade B2.2 was predominantly made of Kenyan sequences. On the global phylogenies (Fig. 4), the major clades observed in A2.2 and B2 clustered separately interspersed with global sequences, suggesting that at least two distinct variants for each subgroup were in circulation. (Fig. 4). Within the clades, the African sequences fell into separate clusters. The most probable location of ancestral sequence at the branches leading to each African clade is indicated next to the nodes for each clade (Fig. 4). The commonly inferred locations included sequences sampled from Malaysia, Peru, Canada and Spain. For A2.1 viruses, African sequences were placed into a single monophyletic clade indicating a single introduction (Additional file 6). Notably, A2.1 sequences were only detected in Zambia and South Africa and clustered closely with sequences from Peru.

### RSV intra country diversity

To assess within-country genetic diversity, Only RSV BA and GA2 viruses were detected in high frequencies across multiple sites and were analysed (Table 2). From the country-specific ML phylogenies, sequences from the different within-country sampling locations were mixed within the phylogenetic clusters suggesting rapid spread movement of RSV variants within each country (Additional file 7). Similarly, the RSV G gene sequences did not cluster by case or control status of the sampled individuals.

### RSV spatial patterns and Origins in Africa

**Fig. 5:**
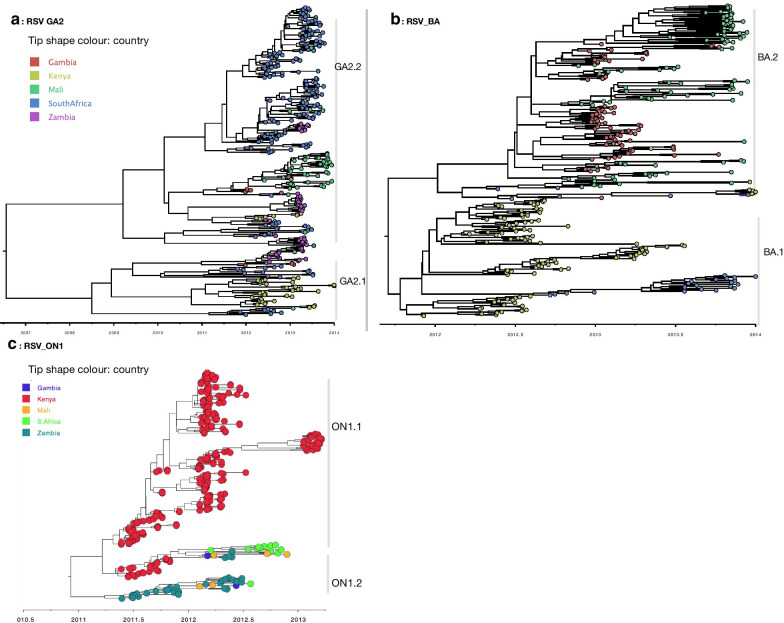
Time-scaled maximum clade credibility trees constructed using RSV GA2 (**a**), BA (**b**) and ON1 (**c**) G gene sequences obtained from Kenya, Mali, Gambia, South Africa and Zambia collected between August 2011 and January 2014. The tips were coloured according to the country of sampling

**Fig. 6:**
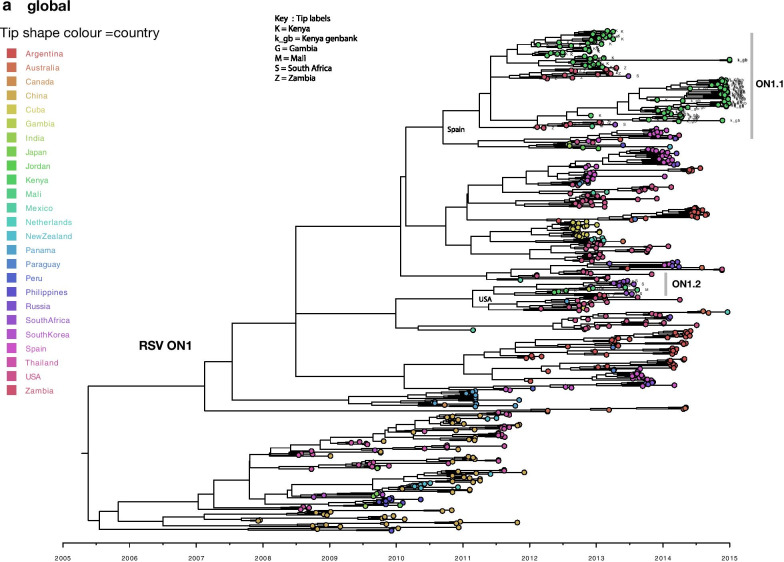
Time-scaled maximum clade credibility tree constructed using RSV ON1 G gene sequences obtained from Africa and GenBank collected between 2010 to 2015. Tree tips are coloured by country of sampling. Geographic locations considered are shown in the figure key. Any sequences from Kenya, Mali, Gambia, South Africa and Zambia obtained from GenBank and collected beyond the study period are indicated with a suffix gb. Clades containing African sequences were labelled to reflect genetic clusters observed on the continental (Africa) ON1 phylogeny—highlighted by grey vertical bars. African sequences are also indicated with tip labels. The most probable location of ancestral sequence at the branches leading to each African monophyletic clade is shown next to the nodes. Only ancestral locations with posterior probability support of > 70% were indicated

RSV phylogeographic analysis revealed markedly similar spatial patterns to those of HMPV. On the continental scale (Africa), geographical clustering was evident, and multiple variants of each RSV genotype were detected (Fig. 5). The inferred continental migration pathways indicated very strongly supported links between neighbouring countries (BF > 1000, posterior probability > 95%) i.e., between The Gambia and Mali, and between South Africa and Zambia (Additional file 8). We further explored the RSV spatial patterns globally to elucidate on the viral introductions into Africa. African ON1 sequences fell into two major clades (numbered ON1.1 and ON1.2, Fig. 6) interspersed with global sequences. Although the clades ON1.1 and ON1.2 were interspersed with global sequences, high sequence similarity (99%) was observed among them indicating widespread movement of similar variants globally. Of the two African clades (Fig. 6), clade ON1.1 clustered closely with sequences from Europe (Spain and Russia) and Asia (India and Jordan). Clade ON1.2 sequences clustered closely to sequences from USA. Similar to ON1, GA2 and BA African sequences were placed into multiple clades alongside global sequences (Additional file 9). Within the clades, GA2 and BA African sequences were interspersed with sequences sampled from different countries globally. Majority of GA2 and BA African sequences clustered with sequences from Thailand, Peru, China, USA, and Spain. Locations of ancestral sequences at the branches leading to each African clade with posterior probability support of > 70% are indicated along the nodes on the phylogenies (Additional file 9).

## Discussion

Our comparative analysis revealed markedly similar patterns of spread of HMPV and RSV within Africa. Geographical clustering of sequences by sub-region was evident with high sequence relatedness between neighbouring countries and separate variant introductions of HMPV and RSV into continental Africa. This observation indicates predominant local transmission and frequently common sources of introduction among neighbouring countries. Within each country, sequences from the different catchment areas were mixed within the phylogenetic clusters, suggesting a rapid movement of HMPV and RSV variants within country upon variant introduction followed by local diversification. However, we cannot ignore the fact that only a single site was sampled in each country. Therefore, we may not have characterised all locally circulating strains. At least two distinct variants of the various genetic groups were observed in each country, indicating multiple importations from the global pool. These results are not unique to only HMPV and RSV as similar findings have been reported for influenza viruses and more recently for SARS-CoV-2 transmission in Kenya, Uganda and South Africa [44–46].

HMPV and RSV epidemics were characterised by co-circulation of multiple genotypes. Genotype circulation patterns were similar between neighboring African countries (South Africa and Zambia, and Mali and The Gambia), indicative of the epidemiological linkage between neighbouring African countries and the independent introduction of multiple HMPV and RSV variants into Africa sub-regions from the global pool. South Africa and Zambia HMPV genotype patterns were characterised by a unique circulation of HMPV A2.1 viruses, which were not detected in the other study sites. On the global phylogenies, HMPV and RSV African sequences were frequently placed into different monophyletic clades interspersed with global sequences, suggestive of multiple sources of virus introduction into African countries and widespread movement of similar variants.

Previous studies of HMPV [47] and RSV [48] done in Argentina reveal the two viruses’ dispersal patterns occur both locally and globally. Similar findings have been reported for influenza viruses in Asia [49] and the USA [50]. Air travel has been shown to be the dominant determinant of influenza H3N2 and H1N1 viruses on the global scale [50, 51]. However, on smaller geographic scales, factors such demography, other forms of mobility, geographical proximity, etc. can be significant predictors of spatial spread [48, 50]. The spatial diffusion pathways of HMPV and RSV revealed strong connections between countries in the same African sub-region and weak links between distant locations. Overall, the patterns of spread of HMPV and RSV observed in this study may reflect underlying host mobility patterns. In particular, Africa experiences separate introduction of HMPV and RSV variants from the global pool influenced by human mobility patterns. Following a virus introduction, there is an establishment of a local epidemic in countries proximal to each other due to more interactions, associated with predominant migration between neighbouring countries [52], as a result of environmental and socioeconomic factors such as distribution of ethnic groups, colonial and regional trade ties [52]. Recent reports on the role of long-distance truck drivers from neighbouring countries on the spread of SARS-CoV-2 in Uganda underscores these links between neighbouring countries [46]. We acknowledge that due to biased sampling, we did not assess possible introductions from unsampled locations. More analysis will be required to test the contribution of human mobility and other potential predictors on the spatial spread to explore the patterns further.

On the global scale, African HMPV and RSV sequences clustered with sequences sampled from different countries, suggesting multiple sources of introduction of HMPV and RSV variants into Africa. African sequences frequently clustered with sequences obtained from Canada, Peru, Malaysia, China, USA and Spain. These links only point to the potential sources of introductions of HMPV and RSV variants into Africa. Future analysis involving representative sampling will help to validate our inferences on the potential sources. Also, due to disproportionate sampling, it was also difficult to pinpoint the main hubs for evolution and selection of HMPV and RSV variants because the discrete trait analysis is inherently biased by the sampling intensities of locations [53, 54]. To pinpoint the key source populations and subsequent sink populations, more representative sampling will be required globally.

Although our analysis was based on a modest sample size (HMPV n = 232 and RSV n = 842 sequences), this did not hinder our ability to assess sequence relatedness and infer spatial-temporal spread of HMPV and RSV in Africa. Also, sequences were collected simultaneously over two years and allowed exploration of the spatial patterns to assess possible epidemiological linkages between Kenya, Mali, Gambia, South Africa, and Zambia. Conversely, we did not assess possible epidemiological links from unsampled locations in Africa. Future studies across different countries in different Africa sub-regions (East, West, South, Central and North) will be necessary for tracing transmission patterns of HMPV and RSV in Africa. Genetic clusters containing similar sequences, especially within-country clusters, will require whole-genome sequencing for increased resolution and detailed transmission studies.

We also analysed the clustering patterns of sequences by cases and controls. Sequences were found not to cluster by the individual’s sampled status. Additionally, we assessed the distribution of HMPV (group A and B) and RSV (group A and B) genetic variants among cases and controls. We found no statistically significant difference in the distribution of cases and controls among the subgroups for both HMPV (pr = 0.873) and RSV (pr = 0.733), Table 3. Our findings show no evidence for differences in disease severity between the subgroups for HMPV and RSV and concur with previous reports [5, 21, 23].

**Table 3:** Sub-group distribution among cases and controls for HMPV and RSV

Group	Case/control		
	Case	Control	Total
*(A) Distribution of HMPV subgroups cases/controls*			
A	84	18	102
	44.21	42.86	43.97
B	106	24	130
	55.79	57.14	56.03
	190	42	232
	100.00	100.00	100.00

Group	Case/control		
	Case	Control	Total
*(B) Distribution of RSV subgroups among cases/controls*			
A	471	38	509
	60.62	58.46	
B	306	27	333
	39.38	41.54	39.55
	777	65	842
	100.00	100.00	100.00

Panel A: Pearson chi2(1) = 0.0256 Pr = 0.873

Panel B: Pearson chi2(1) = 0.1166 Pr = 0.733

The first row shows frequencies, and the second row shows column percentages

Distribution of HMPV and RSV genetic groups determined from sequences collected between August 2011 and January 2014 from the 5 African countries. Panel A: Distribution of HMPV group A and B among cases and controls. Panel B: Distribution of RSV group A and B among cases and controls

HMPV, human metapneumovirus; RSV, respiratory syncytial virus

N/B: There is no statistical significance difference in distribution of cases and control among the subgroups for both HMPV and RSV

## Conclusions

In conclusion, our study provides the first contemporaneous HMPV and RSV sequences across 5 African countries, acting as a significant reference for future molecular epidemiological studies. HMPV and RSV molecular epidemiological patterns were consistent across the study locations in the continent. Multiple strains can co-circulate, and distinct strains can circulate in different Africa sub-regions at the same time. The occurrence of strong regional links suggested that local, tailored public health intervention measures should be considered. By comparing the strain epidemiology geographic patterns of HMPV and RSV across Africa, our study illuminates on the spread characteristics of two seasonally recurring respiratory viruses.

## Supplementary Information

**Additional file 1**: The map of Africa showing the countries and locations from which the sequences were collected. A single site was enrolled in each country i.e. Kilifi; Kenya, Lusaka; Zambia, Bamako; Mali, Soweto; South Africa and Basse; The Gambia [30].

**Additional file 2**: HMPV G and RSV G genes PCR and sequencing primers used to generate the sequence data reported in this study.

**Additional file 3**: The GenBank accession numbers of HMPV and RSV attachment (G) glycoprotein gene sequences generated in this study and the contemporaneous sequences retrieved from GenBank. Sheet1: RSV accession numbers grouped by African country of sampling and global sequences. Sheet2: HMPV accession numbers grouped by African country and global sequences.

**Additional file 4:** ML phylogenies of HMPV and RSV G gene sequences collected from Kenya, Mali, Gambia, South Africa and Zambia. Sequences were subtyped based on clustering with with known subgroups or prototype sequences of HMPV and RSV retrieved from GenBank. Panel a: HMPV G gene sequences constructed using 231G gene sequences. Prototype sequences are coloured in red. The numbers next to branches indicate the bootstrap values. Subgroups were confirmed if sequences clustered with known subgroup-specific sequences within a major branch with > 70% bootstrap support. Panel b: RSV G ML phylogeny constructed using 627 unique gene sequences.

**Additional file 5:** ML phylogenies of HMPV subgroup B1 sequences showing within country sequence diversity for Kenya, Gambia, Mali and South Africa sequences. Clustering patterns were determined by within-country sampling location (left panel) and or case/control status (right panel). For Gambia, only case/control clustering patterns were determined.

**Additional file 6:** Panel A; Time-scaled maximum clade credibility (MCC) tree constructed using HMPV A2.1 G gene sequences obtained from Africa and GenBank collected between 2000 to 2018. Branches are coloured according to the most probable location as inferred using symmetric discrete phylogeographic diffusion model. Geographic locations considered are shown in the figure key. Posterior probabilities are shown next to nodes. Clades containing African sequences falling in monophyletic clades are highlighted by coloured tip labels. Panel B; time scaled MCC tree of HMPV A2.1 sequences collected from Africa.

**Additional file 7:**ML phylogenies of RSV BA and GA2 sequences showing within country sequence diversity for Mali and South Africa sequences who’s within-country sampling information was available. Clustering patterns were determined by within-country sampling location (left panel) and or case/control status (right panel).

**Additional file 8**: Statistically supported state transitions indicating viral migration events between African countries. Bayes factor > 100 and Posterior probability ≥ 95% was considered significant.

**Additional file 9:** Time-scaled maximum clade credibility (MCC) trees constructed using RSV GA2 (Panel A) and RSV B2 (Panel B) G gene sequences obtained from Africa and GenBank collected between 2010 to 2015. Tree tips are coloured by country of sampling. Geographic locations considered are shown in the figure key. Any sequences from Kenya, Mali, Gambia, South Africa and Zambia obtained from GenBank and collected beyond the study period are indicated with a suffix _gb. African sequences are indicated with tip labels. The most probable location of ancestral sequence at the branches leading to each African monophyletic clade is shown next to the nodes. Only ancestral locations with posterior probability support of > 70% were indicated.

## Data Availability

The replication data set for this manuscript are available from the Harvard Dataverse under the doi: https://dataverse.harvard.edu/dataset.xhtml?persistentId=doi:10.7910/DVN/POHLE3. Data access can be requested from the KEMRI-Wellcome Trust Research Programme, Data Governance Committee (dgc@kemri-wellcome.org). Publicly accessible data are included in this published article (Additional file 1).

## Abbreviations

HMPV: Human metapneumovirus
RSV: Respiratory syncytial virus
ALRTI: Acute lower respiratory tract infection
tMRCA: Time to the most recent common ancestor
PERCH: Pneumonia Etiology Research for Child Health
ESS: Effective sample size
KML: Keyhole markup language
BSSVS: Bayesian Stochastic Search Variable Selection
