# Supplementary figures and images for: Comparative analysis of spatial-temporal patterns of human metapneumovirus and respiratory syncytial virus in Africa using genetic data, 2011–2014
Source: Virol J. 2021 May 29;18:104. doi: 10.1186/s12985-021-01570-8 (PMC8164071; doi:10.1186/s12985-021-01570-8)

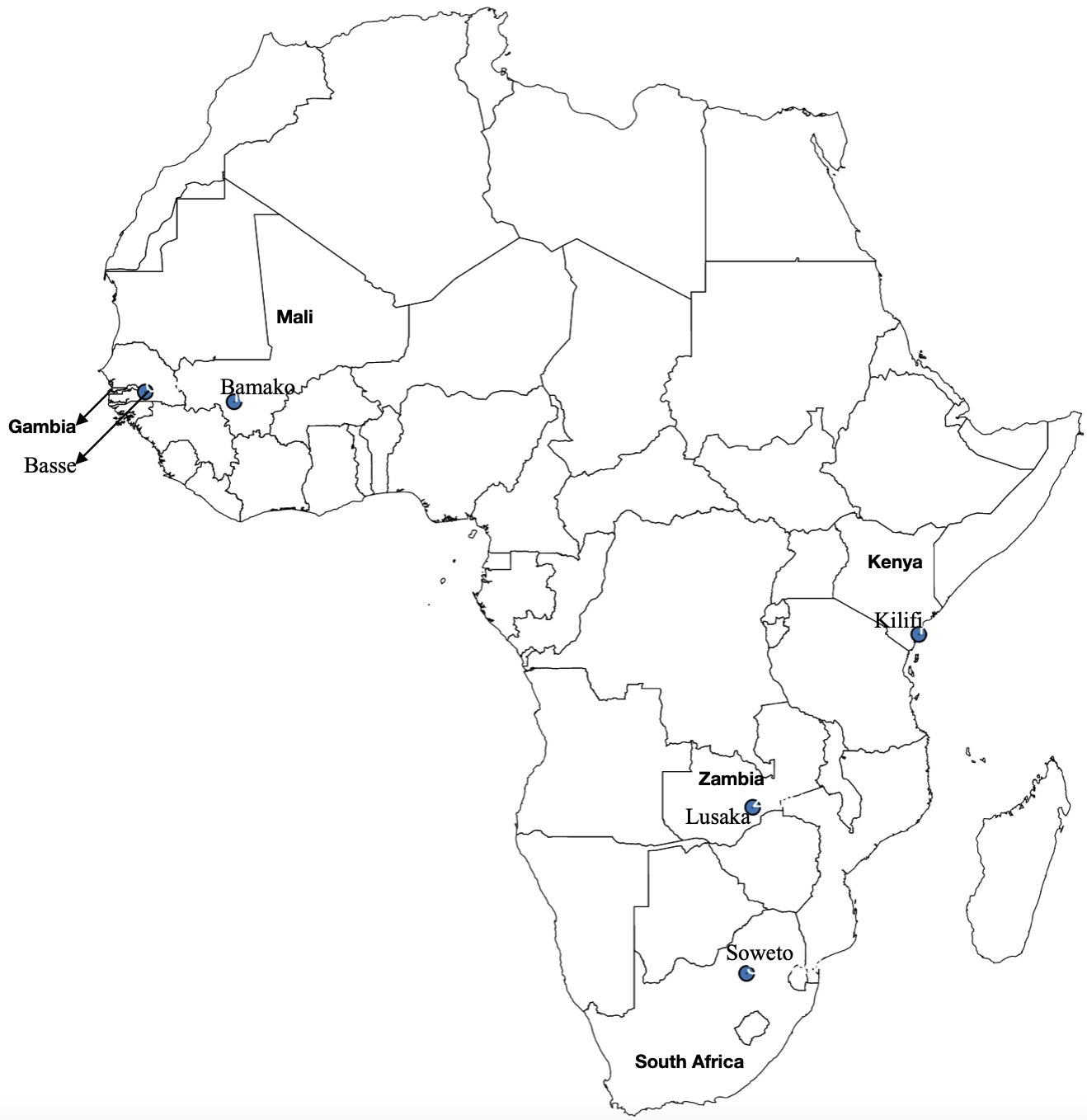

Supplement: Supplementary file 1 — Additional file 1: The map of Africa showing the countries and locations from which the sequences were collected. A single site was enrolled in each country i.e. Kilifi; Kenya, Lusaka; Zambia, Bamako; Mali, Soweto; South Africa and Basse; The Gambia [30]. [file 12985_2021_1570_MOESM1_ESM.png]

### Stratified by case/control status

**E**

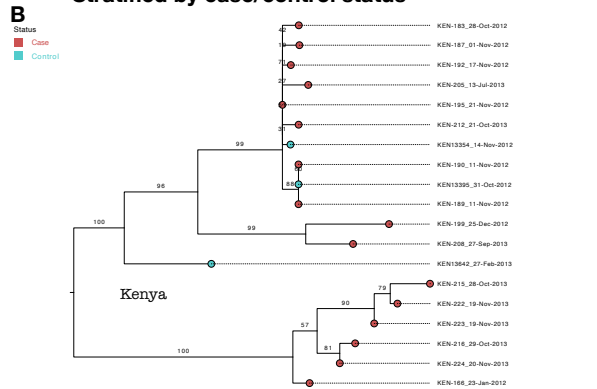

D

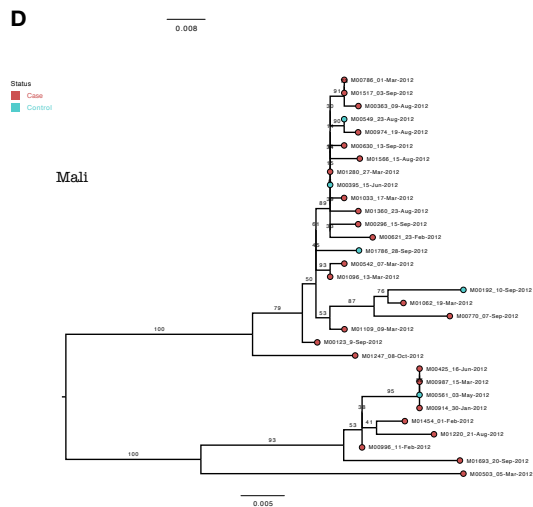

**F**

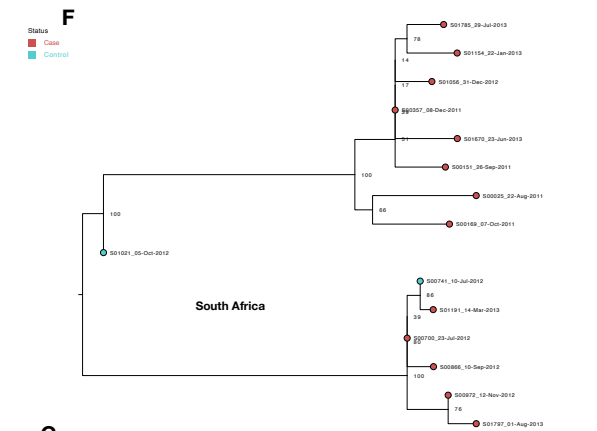

## G

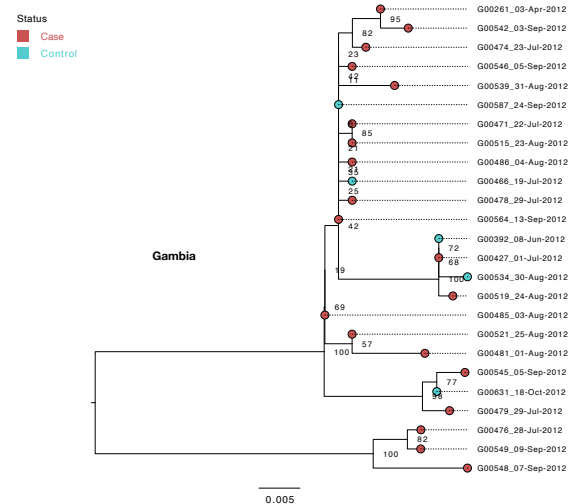

Supplement: Supplementary file 5 — Additional file 5: ML phylogenies of HMPV subgroup B1 sequences showing within country sequence diversity for Kenya, Gambia, Mali and South Africa sequences. Clustering patterns were determined by within-country sampling location (left panel) and or case/control status (right panel). For Gambia, only case/control clustering patterns were determined. [file 12985_2021_1570_MOESM5_ESM.pdf]

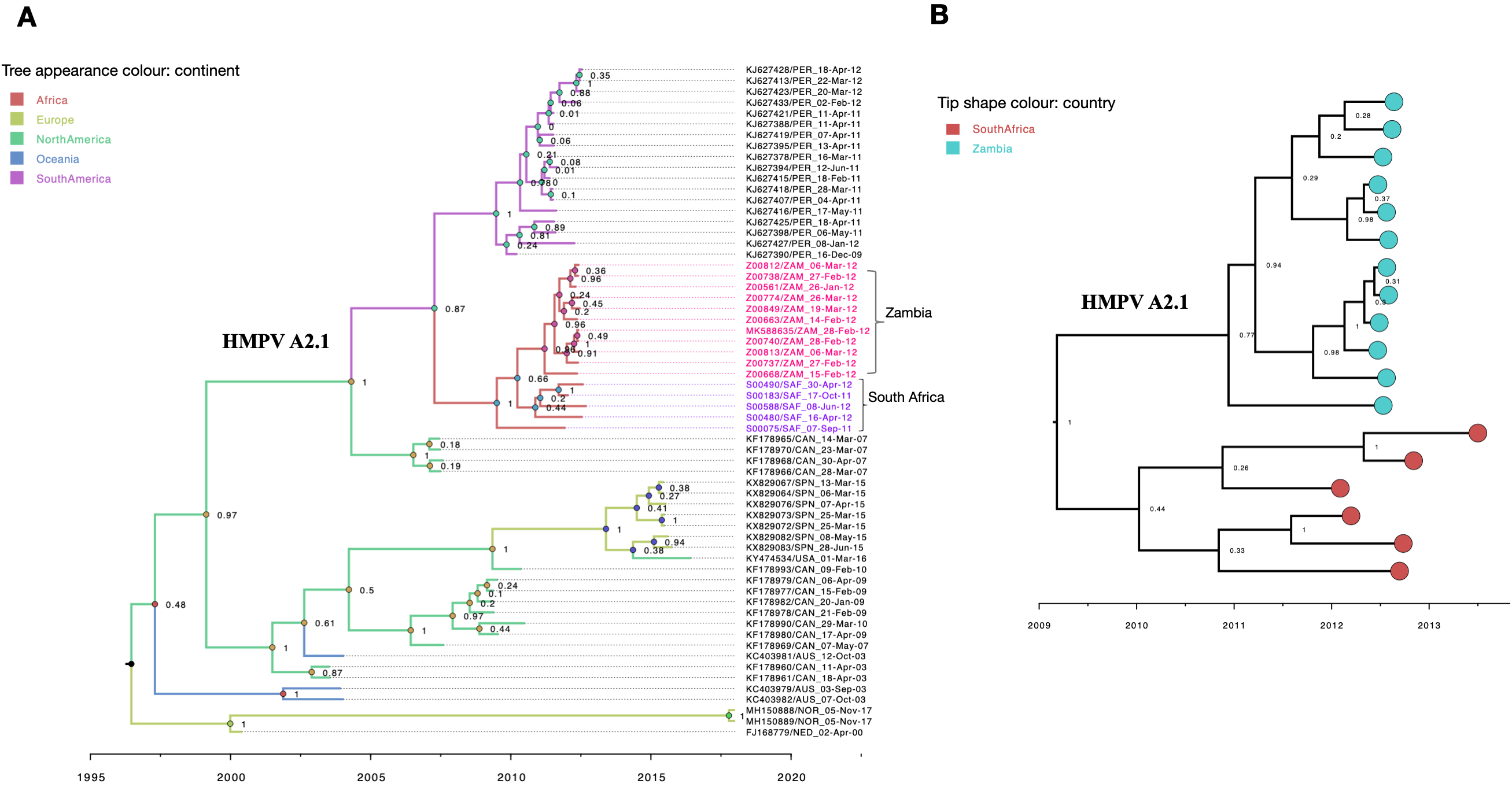

Supplement: Supplementary file 6 — Additional file 6: Panel A; Time-scaled maximum clade credibility (MCC) tree constructed using HMPV A2.1 G gene sequences obtained from Africa and GenBank collected between 2000 to 2018. Branches are coloured according to the most probable location as inferred using symmetric discrete phylogeographic diffusion model. Geographic locations considered are shown in the figure key. Posterior probabilities are shown next to nodes. Clades containing African sequences falling in monophyletic clades are highlighted by coloured tip labels. Panel B; time scaled MCC tree of HMPV A2.1 sequences collected from Africa. [file 12985_2021_1570_MOESM6_ESM.png]

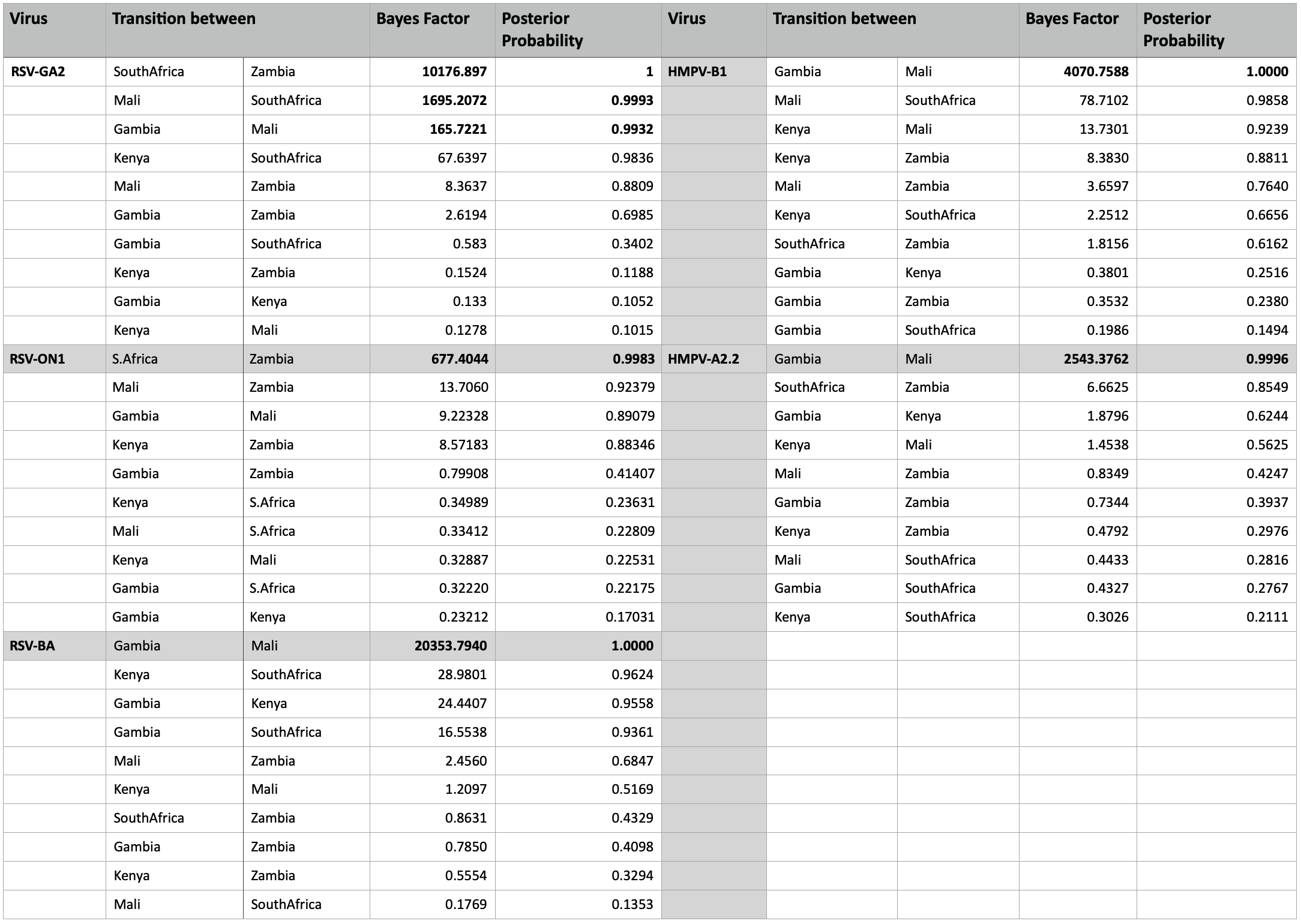

Supplement: Supplementary file 8 — Additional file 8: Statistically supported state transitions indicating viral migration events between African countries. Bayes factor > 100 and Posterior probability ≥ 95% was considered significant. [file 12985_2021_1570_MOESM8_ESM.png]

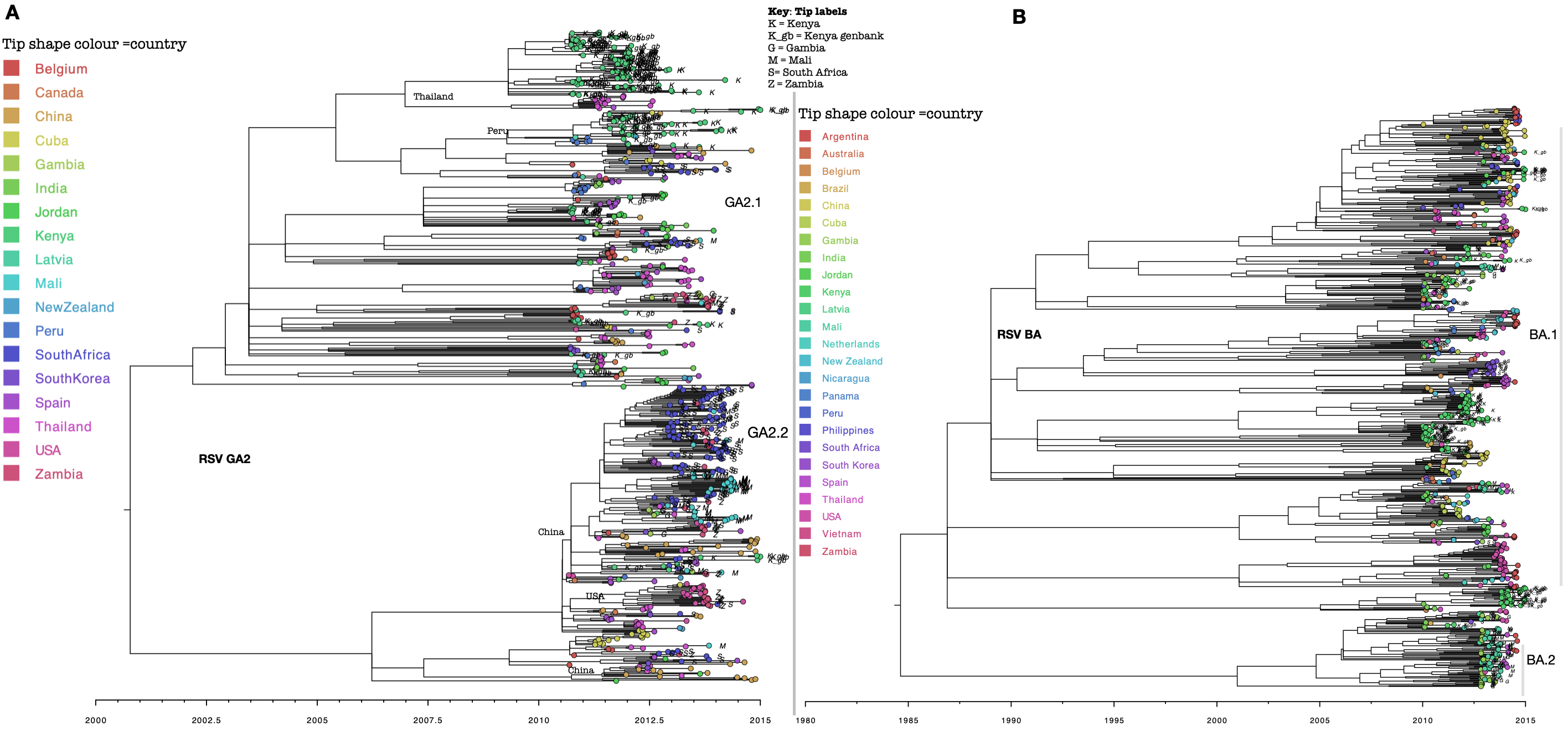

Supplement: Supplementary file 9 — Additional file 9: Time-scaled maximum clade credibility (MCC) trees constructed using RSV GA2 (Panel A) and RSV B2 (Panel B) G gene sequences obtained from Africa and GenBank collected between 2010 to 2015. Tree tips are coloured by country of sampling. Geographic locations considered are shown in the figure key. Any sequences from Kenya, Mali, Gambia, South Africa and Zambia obtained from GenBank and collected beyond the study period are indicated with a suffix _gb. African sequences are indicated with tip labels. The most probable location of ancestral sequence at the branches leading to each African monophyletic clade is shown next to the nodes. Only ancestral locations with posterior probability support of > 70% were indicated. [file 12985_2021_1570_MOESM9_ESM.png]
