# Supplementary material for: Comparative analysis of spatial-temporal patterns of human metapneumovirus and respiratory syncytial virus in Africa using genetic data, 2011–2014
Source: Virol J. 2021 May 29;18:104. doi: 10.1186/s12985-021-01570-8 (PMC8164071; doi:10.1186/s12985-021-01570-8)
Supplement: Supplementary file 2 — Additional file 2: HMPV G and RSV G genes PCR and sequencing primers used to generate the sequence data reported in this study. [file 12985_2021_1570_MOESM2_ESM.docx]

## Appendix A

Table A1: HMPV G and RSV G genes PCR and sequencing primers

|  | **Primer name** |  | **Sequence** | **Gene** | **Organism** | **Polarity** | **Subgroup** |
| --- | --- | --- | --- | --- | --- | --- | --- |
| **1** | AG20 F | 1^st^ round PCR | GGGGCAAATGCAAACATGTCC | G | RSV | + | AB |
| **2** | F164 R | 1^st^ round PCR | GTTATGACACTGGTATAC CAACC | G | RSV | - | AB |
| **3** | BG10 F | 2^nd^ round PCR | GCAATGATAATCTCAACCTC | G | RSV | + | AB |
| **4** | F1 R | 2^nd^ round PCR | CAACTCCATTGTTATTTGCC | G | RSV | - | AB |
| **5** | G523 F | Sequencing | ATATG CAGCAACAATCCAAC | G | RSV | + | A |
| **6** | G523 R | Sequencing | GTTG GATTGTTGCTGCATAT | G | RSV | - | A |
| **7** | G533 F | Sequencing | TGTAGTATATGTGGCAACAA | G | RSV | + | B |
| **8** | G533 R | Sequencing | TTGTTGCCACATATACTACA | G | RSV | - | B |
| **9** | 13F | PCR + sequencing | GTRGAGAACATTCGAGCAATAGACA | G | HMPV | + | A |
| **10** | 264F | Sequencing | TCCAAACTCACAGCATCCAAC | G | HMPV | + | A |
| **11** | 1163R | PCR + sequencing | AGGGAGATAGACATTAACAGTGGA | G | HMPV | - | A |
| **12** | 2F | PCR + sequencing | TGGAAGTAAGAGTGGAGAACATTC | G | HMPV | + | B |
| **13** | 222F | Sequencing | YAARAAGACCCCAATGACCTC | G | HMPV | + | B |
| **15** | 718R | Sequencing | ACTACTTGGATGAGATACCTGTGT | G | HMPV | - | B |
| **16** | 1098R | PCR + sequencing | TGACTGCATTTCTAAGCCTTACAT | G | HMPV | - | B |
