# Supplementary material for: Comparative analysis of spatial-temporal patterns of human metapneumovirus and respiratory syncytial virus in Africa using genetic data, 2011–2014
Source: Virol J. 2021 May 29;18:104. doi: 10.1186/s12985-021-01570-8 (PMC8164071; doi:10.1186/s12985-021-01570-8)
Supplement: Supplementary file 4 — Additional file 4: ML phylogenies of HMPV and RSV G gene sequences collected from Kenya, Mali, Gambia, South Africa and Zambia. Sequences were subtyped based on clustering with with known subgroups or prototype sequences of HMPV and RSV retrieved from GenBank. Panel a: HMPV G gene sequences constructed using 231G gene sequences. Prototype sequences are coloured in red. The numbers next to branches indicate the bootstrap values. Subgroups were confirmed if sequences clustered with known subgroup-specific sequences within a major branch with > 70% bootstrap support. Panel b: RSV G ML phylogeny constructed using 627 unique gene sequences. [file 12985_2021_1570_MOESM4_ESM.pdf]

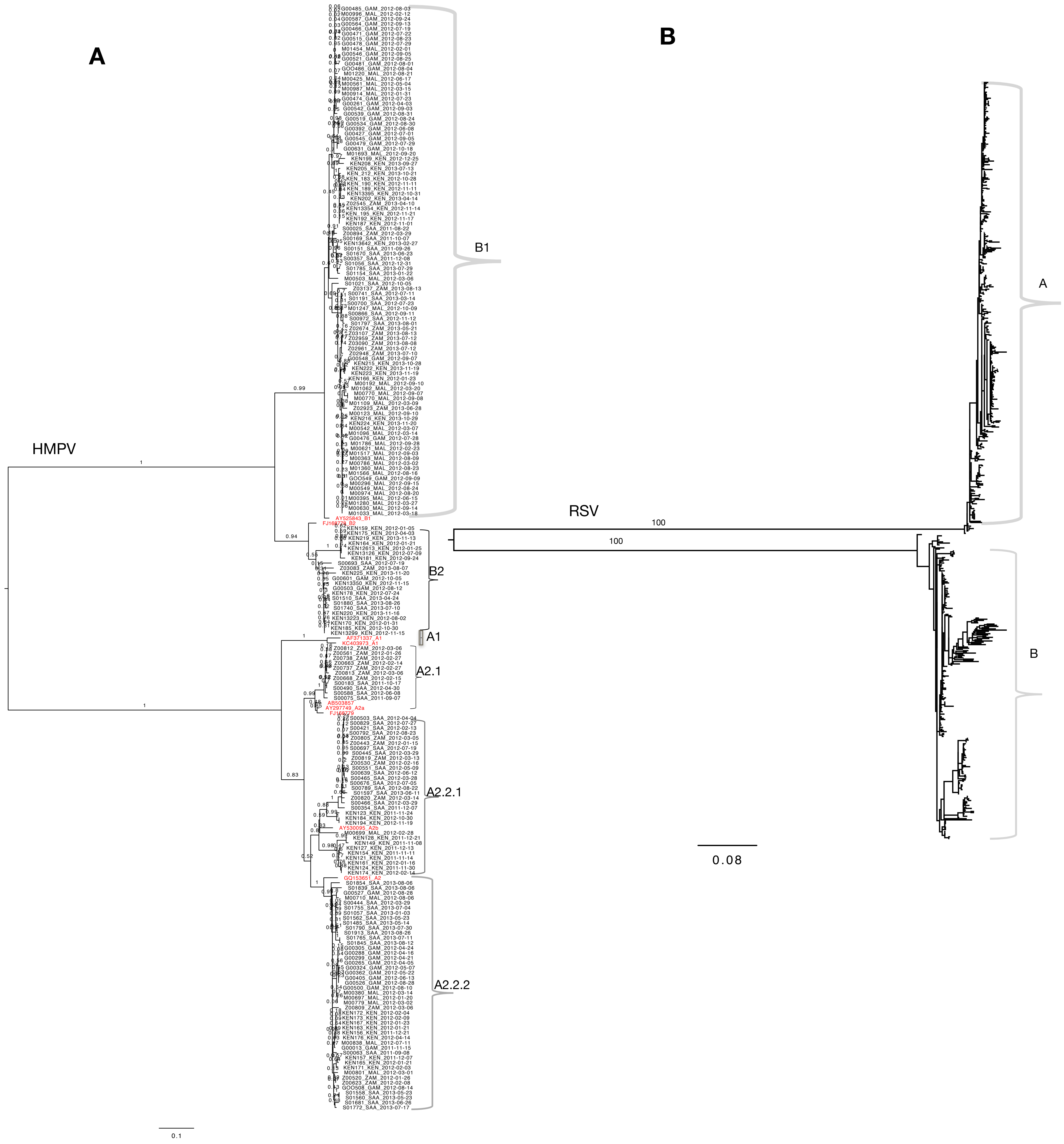

A
HMPV
B1
B2
A1
A2.1
A2.2.1
A2.2.2
0.1
B
RSV
100
100
A
B
0.08
