# Supplementary material for: Comparative analysis of spatial-temporal patterns of human metapneumovirus and respiratory syncytial virus in Africa using genetic data, 2011–2014
Source: Virol J. 2021 May 29;18:104. doi: 10.1186/s12985-021-01570-8 (PMC8164071; doi:10.1186/s12985-021-01570-8)
Supplement: Supplementary file 7 — Additional file 7:ML phylogenies of RSV BA and GA2 sequences showing within country sequence diversity for Mali and South Africa sequences who’s within-country sampling information was available. Clustering patterns were determined by within-country sampling location (left panel) and or case/control status (right panel). [file 12985_2021_1570_MOESM7_ESM.pdf]

RSV BA

A Stratified by location

B Stratified by case/control status

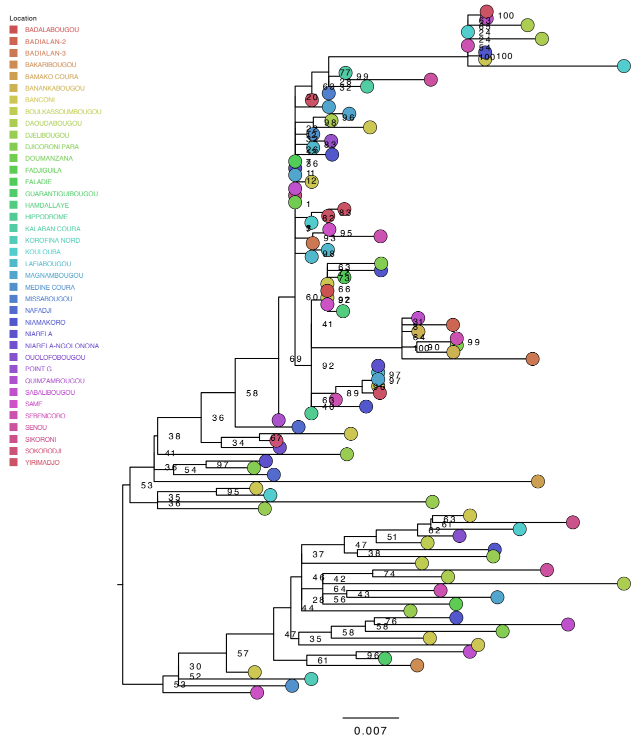

Mali

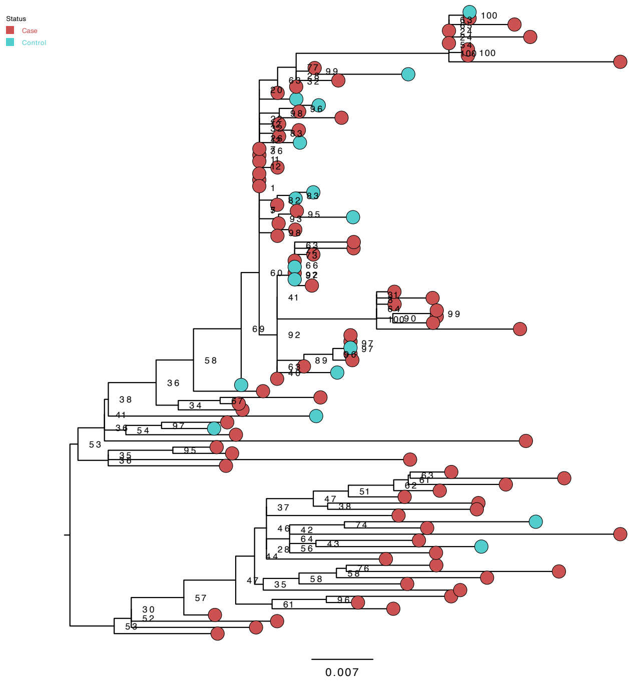

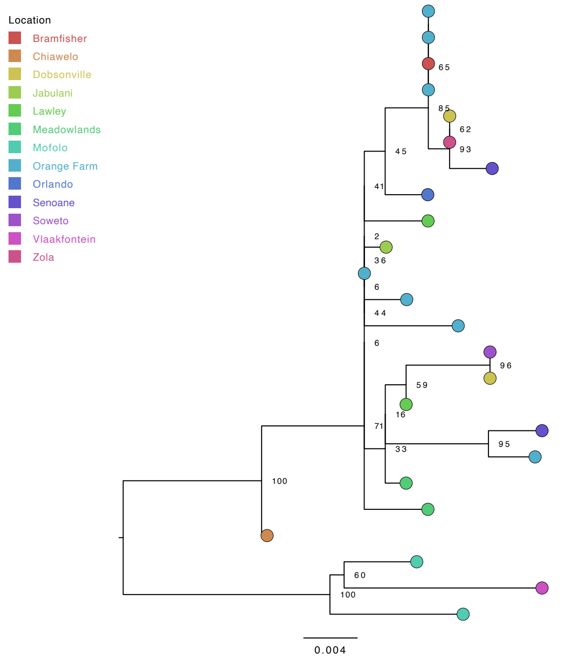

South Africa

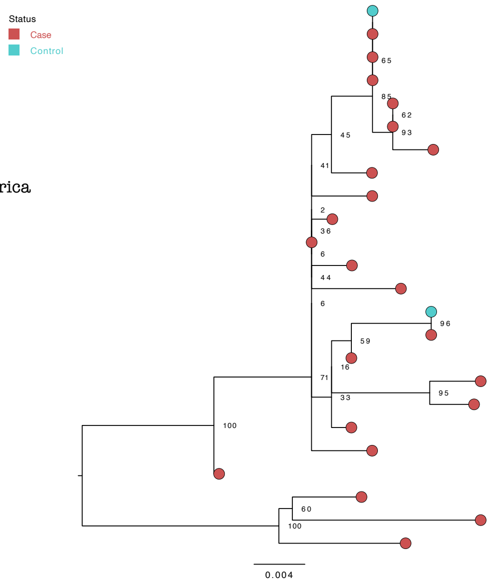

RSV GA

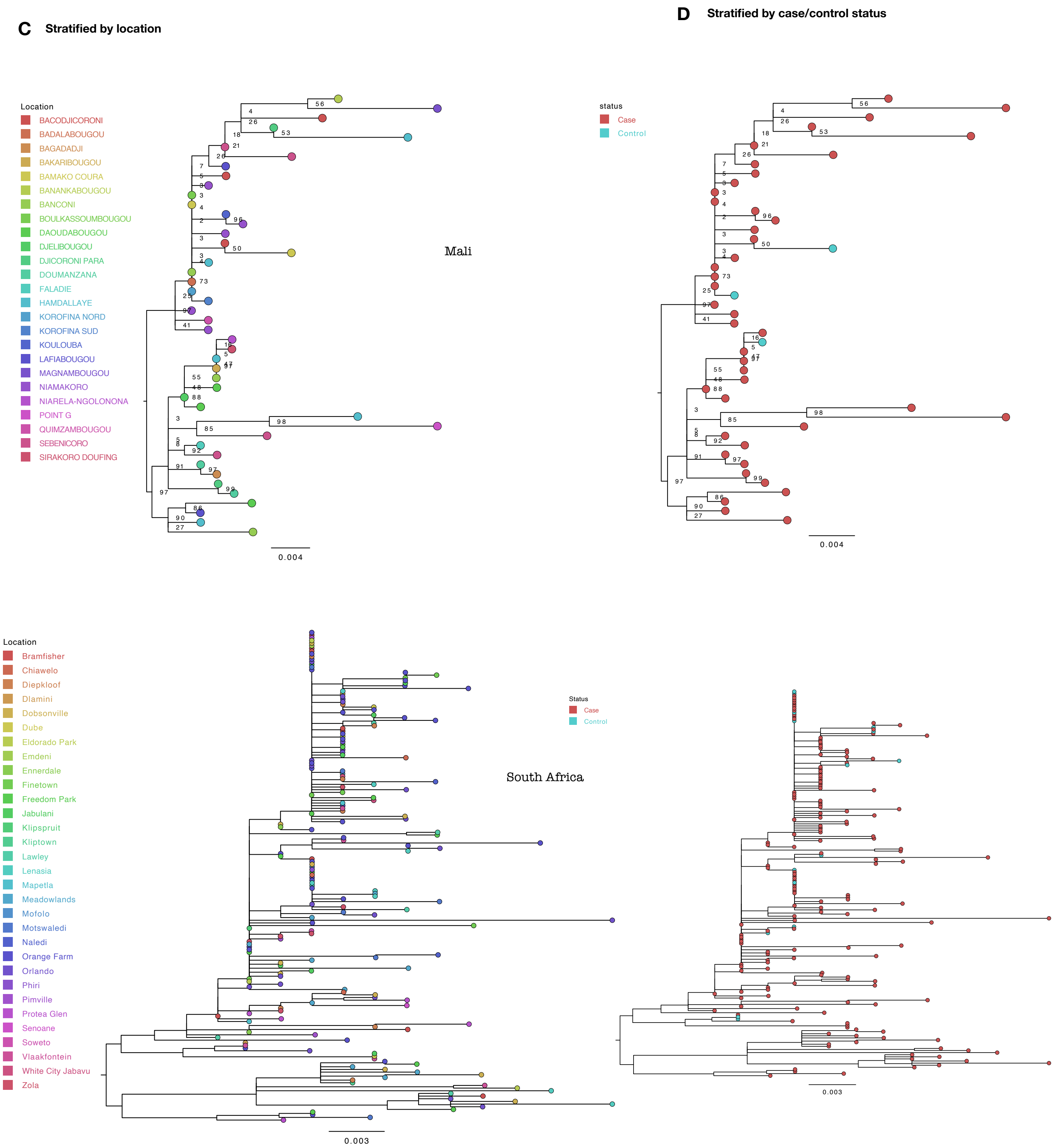
C Stratified by location
D Stratified by case/control status
Location
BACODJICORONI
BADALABOUGOU
BAGADADJI
BAKARIBOUGOU
BAMAKO COURA
BANANKABOUGOU
BANCONI
BOULKASSOUMBOUGOU
DAOUDABOUGOU
DJELIBOUGOU
DJICORONI PARA
DOUMANZANA
FALADIE
HAMDALLAYE
KOROFINA NORD
KOROFINA SUD
KOULOUBA
LAFIABOUGOU
MAGNAMBOUGOU
NIAMAKORO
NIARELA-NGOLONONA
POINT G
QUIMZAMBOUGOU
SEBENICORO
SIRAKORO DOUFING
Mali
0.004
status
Case
Control
0.004
Location
Bramfisher
Chiawelo
Diepkloof
Dlamini
Dobsonville
Dube
Eldorado Park
Emdeni
Ennerdale
Finetown
Freedom Park
Jabulani
Klipspruit
Kliptown
Lawley
Lenasia
Mapetla
Meadowlands
Mofolo
Motswaledi
Naledi
Orange Farm
Orlando
Phiri
Pimville
Protea Glen
Senoane
Soweto
Vlaakfontein
White City Jabavu
Zola
South Africa
0.003
Status
Case
Control
0.003
